# A Hint for Hospital Secretaries

**Published:** 1908-09-26

**Authors:** 


### A HINT FOR HOSPITAL SECRETARIES.

The way in which the Guest Hospital came to inherit Mr. Henry Lewis's fortune of £90,000 conveys a suggestion to hospital secretaries, which it is to be hoped they will profit by. Mr. Bird, the secretary of the hospital, learned six years ago that Mr. Lewis proposed to bequeath a considerable sum to a Methodist Chapel, and fancied that a man who possesses such generous instincts would be willing to help a hospital. Therefore, he sent Mr. Lewis a statement of the financial position of the institution, showing the need of funds. So far as Mr. Bird knew the appeal failed—at least Mr. Lewis did not reply. But immediately thereafter an anonymous gift of bank-notes to the value of £500 came to him. Then an anonymous donor sent books, pianos, and other things to the hospital in generous measure. Finally, Mr. Lewis's death revealed the truth—that he had been the unknown giver, and that in addition he had bequeathed his whole fortune to the hospital. The secretary's appeal, which apparently had met with no response, had really worked marvels. The incident accentuates the fact, often imperfectly realised, that upon the energy of the secretary the financial position of a charitable institution largely depends. He must be instant in season and out of season, and be deterred by no rebuffs. No doubt many of his appeals for help will be fruitless, but against these must be set those which bring in a generous harvest. Nor must he be discouraged because the response is not immediate. In the case of Mr. Lewis it was prompt, but secret, and Mr. Bird may well have thought that he had asked in vain. In other cases it may really be neglected at the moment, but at some later time—say when the person appealed to has had personal experience of illness—it may be recalled and acted upon. But, of course, the first essential is a good case for the appeal—not a mere vague talk, but a straight, financial statement. Business men—and it is to business men that all institutions must look for their largest donations—like business methods. And this, too, is in the secretary's hands. He must be sure that the institution he is connected with can bear inspection as to its working and expenditure, and then he must be able to put its need plainly before those he asks for help.

